# Investigation on the Short-Term Aging-Resistance of Thermoplastic Polyurethane-Modified Asphalt Binders

**DOI:** 10.3390/polym10111189

**Published:** 2018-10-25

**Authors:** Ruien Yu, Xijing Zhu, Maorong Zhang, Changqing Fang

**Affiliations:** 1 School of Mechanical Engineering, North University of China, Taiyuan 030051, China; zxj161501@nuc.edu.cn (X.Z.); zhangmaorong@nuc.edu.cn (M.Z.); 2 Shanxi Key Laboratory of Advanced Manufacturing Technology, North University of China, Taiyuan 030051, China; 3 School of Mechanical and Precision Instrument Engineering, Xi’an University of Technology, Xi’an 710048, China; fangcq@xaut.edu.cn

**Keywords:** modified-asphalt, thermoplastic polyurethane, rheological properties, thermal properties, microstructure

## Abstract

In this reported work, thermoplastic polyurethane (TPU) was used as a reactive polymer modifying agent to prepare a modified-asphalt, using a high-speed shearing method. Physical performance tests of the TPU-modified asphalt were conducted before and after short-term aging, and the aging resistance was examined by the change in materials properties. In addition, low-temperature rheological properties, thermal properties, the high-temperature storage stability, and the aging mechanism of TPU-modified asphalt were also investigated. The results showed that the addition of TPU improved the aging resistance of base asphalt, which was evidenced by the increased penetration ratio and decreased softening point of the asphalt, after aging. Similarly, Fourier Transform infrared (FTIR) spectroscopy results verified that TPU improved the asphalt aging resistance. It was found that the TPU functional groups played a role in improving thermal properties, high-temperature storage stability, and in the dispersion of modified asphalt.

## 1. Introduction

Asphalt is an engineering material used in many industrial sectors of the national economy. Its wide range of uses makes asphalt an irreplaceable product, particularly in road construction and in waterproofing of buildings [1]. However, increased road traffic and the heavier vehicle loads demand that the requirements for road construction is greatly improved, which translates to improving the properties of asphalt materials [2,3]. The elastomer rubber was the earliest asphalt modifier and rubber-modified asphalt offer advantages in terms of low-temperature cracking resistance, elastic properties, and toughness, while reducing traffic noise to improve driving comfort [4,5]. Combining the properties of both rubber and polymer resins, styrene butadiene styrene (SBS) can comprehensively improve the properties of base asphalt and, currently, become the most used, and studied, asphalt modifier [6,7,8,9]. While additives help to improve the performance of asphalt, they also create some problems, such as the compatibility of the modifier and the base asphalt [10], stability of the modified-asphalt [11], and the balance between high- and low-temperature properties [12]. To solve these problems, many researchers added nanomaterials into base asphalt or polymer-modified asphalt (PMA) to make up for the deficiencies of PMA in performance. Polacco et al. [13], Zhang et al. [14,15] and other scholars believe that the exfoliated and intercalated structure is formed in a nano-layered material/polymer/asphalt system. They also believe that these structures, especially the exfoliated structure, can separate oxygen and prevent the volatilization of the light-asphalt components, thereby, increasing the aging resistance of asphalt and improving the service life of a modified asphalt binder.

Nanomaterials-modified asphalt technology has made some progress, such as aging resistance, rheological properties, and high-temperature performance. However, due to the complex composition, and viscosity features of asphalt, combined with a huge surface area and high surface energy of nanoparticles, the agglomeration phenomenon of nanomaterials is ubiquitous in a nano-modified asphalt. In addition, the cost of nanomaterials can also limit its engineering application. The thermoplastic polyurethane (TPU) contains many carbamate groups (–NHCOO–), in the main chain, as a typical multi-block copolymer [16]. While improving the performance of asphalt, its compatibility can also be improved by the reaction between the functional groups, in the TPU and the components of the base asphalt [17]. Partal et al. [18,19,20] have extensively investigated polyurethane prepolymer and isocyanate-based polymer-modified asphalt. TPU is a kind of polymer, with both rigid and flexible properties, as its molecule contains hard and soft segments. As an asphalt modifier, the TPU can not only improve the strength but also the flexibility of the modified asphalt system [21]. Actually, the present literature is lacking in the reporting of TPU-modified asphalt binders. The present study focused on the physical performance, as well as low-temperature rheological properties of TPU-modified asphalt. In addition, the thermal properties, the high-temperature storage stability of TPU-modified asphalt and its aging mechanism were also studied.

## 2. The Experimental Process

### 2.1. Materials

Base asphalt 90A was obtained from Xi’an Petroleum & Chemical Corporation (Xi’an, China). Commercial TPU (Desmopan 9380A) was obtained from Bayer AG (Werk Leverkusen, Germany), with a density of 1.110 g/cm^3^, and a number-average molecular weight of about 100,000. TPU easily absorbs moisture so the resin was thoroughly dried, before use.

### 2.2. Sample Preparations

A FLUKO FM300 high shear emulsifier (Shanghai, China) was used to mix the TPU and the base asphalt. The mixing process began with heating 500 g of the base asphalt, until it became pourable. Subsequently, specific quantities of TPU were individually added into the melted asphalt and the mixture was sheared at 150 °C, for 1 h, with a speed of 2500 rpm. Finally, the binders were stirred with a mechanical agitator for another 1 h, in 150 °C. We prepared a series of compositions with 1 wt %, 2 wt %, 3 wt %, and 4 wt % of TPU contents, based on the weight of the base asphalt.

### 2.3. Aging Procedure

Short-term aging of the base asphalt and the modified-asphalts was conducted, using a rolling thin-film oven test (RTFOT), according to American Society for Testing Materials (ASTM) D2872.

### 2.4. Tests Procedures

#### 2.4.1. Physical Properties Test

The performance indexes of asphalt samples, such as penetration, softening point, and ductility (5 °C) were obtained from tests conducted in accordance with ASTM D5, ASTM D36, and ASTM D113, respectively. The penetration ratio, softening point increment, and ductility ratio of the asphalt samples before and after aging were used to evaluate the aging resistance of the materials.

#### 2.4.2. Low-Temperature Creep Test

Low-temperature creep tests were performed using a bending beam rheometer (BBR, Cannon Instrument Company, State College, PA, USA), in accordance with ASTM D6648. The creep stiffness (S) and creep rate (m) of the asphalt samples were tested at –24 °C and –18 °C.

#### 2.4.3. Thermal Gravity Test

Thermal gravity test was carried out using a TGA/DSC 1 thermogravimetric analyzer (Mettler Toledo, Zurich, Switzerland), under an air atmosphere, at a scan rate of 15 °C/min, from 100 to 700 °C.

#### 2.4.4. Storage Stability Test

Modified asphalt sample was transferred into a glass tube and stored vertically in an oven, at 163 °C. After 48 h, the tube was removed, cooled to room temperature and then the sample was cut into three equal sections, horizontally. The top and bottom sections were used to evaluate the storage stability of the modified-asphalt, by measuring the softening points. The test was conducted in accordance with ASTM D5976.

#### 2.4.5. Morphology Observation

The sample’s morphology was observed using a Nikon 80i fluorescent microscope (Tokyo, Japan). A small amount of the heated asphalt sample was placed on a glass microscope slide and then pressed into a thin layer with a cover glass. After cooling, the sample was inspected at a magnification of 400 times, under a green incident light.

#### 2.4.6. FTIR Test

Infrared spectra of the samples were recorded using a Shimadzu FTIR-8400S spectrometer (Kyoto, Japan), with scans conducted from 400 to 4000 cm^−1^, with a 4 cm^–1^ resolution. Asphalt samples were individually dissolved in tetrahydrofuran and the solution was painted onto a potassium bromide (KBr) thin plate, for testing.

## 3. Results and Discussion

### 3.1. Physical Properties

**Figure 1:**
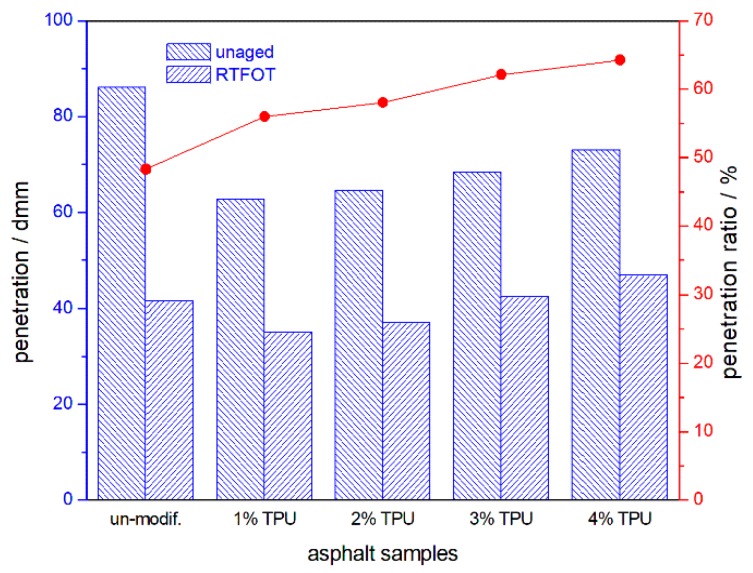
Penetration of the asphalt samples and their residual penetration ratio, after the RTFOT aging.

**Figure 2:**
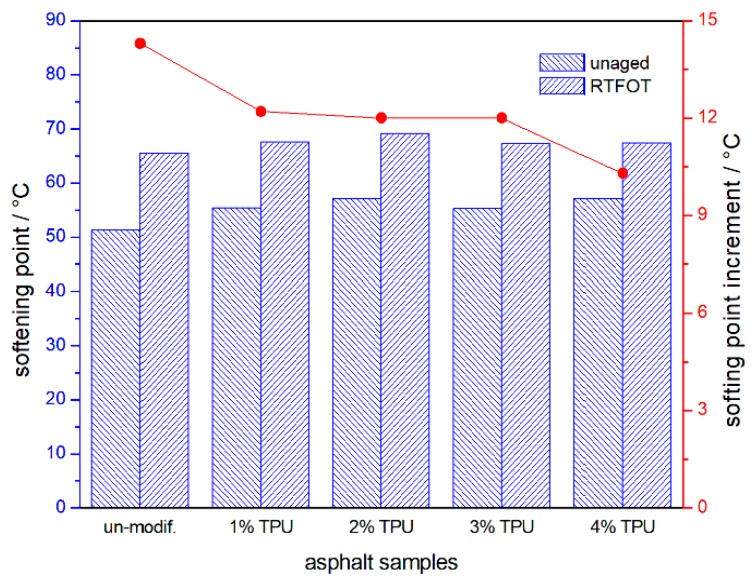
Softening point of the asphalt samples and their increment, after the RTFOT aging.

**Figure 3:**
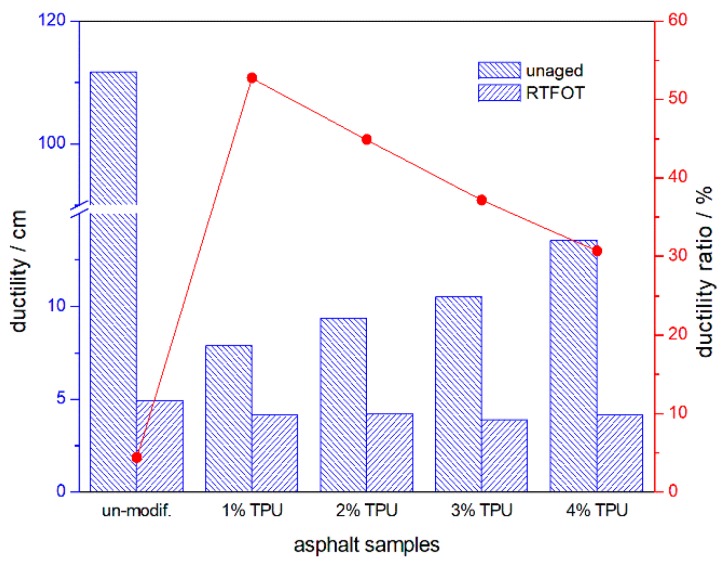
Ductility of the asphalt samples and their residual ductility ratio, after the RTFOT aging.

The penetration, softening point, and ductility of the asphalt samples and their ratio or increment, before and after aging, are shown in Figure 1, Figure 2 and Figure 3 respectively. Compared with the base asphalt in Figure 1, the penetration of the TPU-modified asphalt sample appeared to be reduced, indicating that the modified-asphalt was toughened and its shear deformation resistance was also enhanced. However, as the TPU content increased, the modified-asphalt became softer. After RTFOT aging, the ratio of residual penetration and the penetration before aging increased and the aging resistance of the asphalt improved. In Figure 2, the TPU-modified asphalts displayed a softening point of 4–6 °C higher than the base asphalt. There was no significant difference between the two, with little improvement in high-temperature performance. With the increase of TPU content, the softening point increment decreased after the RTFOT aging, demonstrating the improved aging resistance of the asphalt. Base asphalt has an excellent ductility, and its low temperature value was 111.7 cm, as shown in Figure 3. However, the light molecular weight (MW) asphalt components, such as saturated and aromatic hydrocarbons were oxidized and volatilized in the following the RTFOT aging, so that the residual ductility was only 4.8% of the original value. This would indicate that, in the field, the brittleness of the asphalt pavement would increase, thereby, decreasing its cracking resistance at a low temperature. In the case of the TPU-modified asphalts, the low temperature ductility increased with an increasing TPU content and the ductility ratio was higher than the base value, but with only a 4 cm of residual ductility, it was still at a very low level.

### 3.2. Low-Temperature Creep Properties

**Figure 4:**
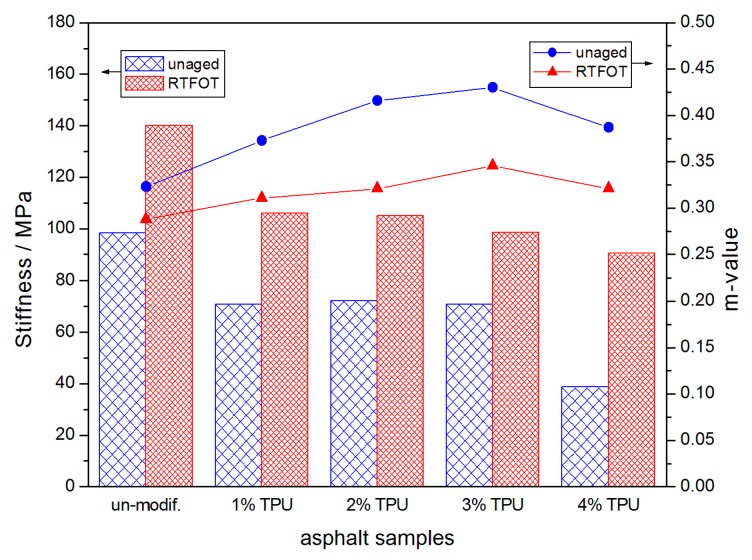
Creep stiffness (S) and creep rate (m-value) of the asphalt samples, before and after the RTFOT aging, at −18 °C.

**Figure 5:**
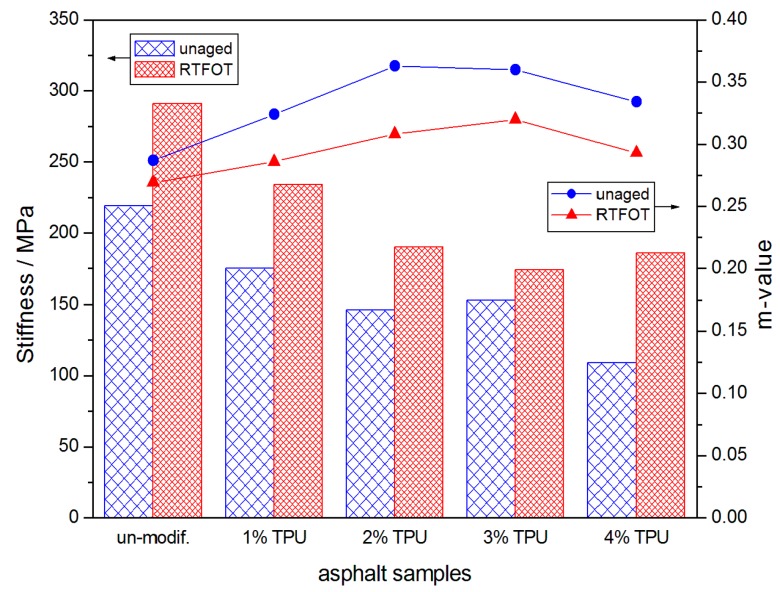
Creep stiffness (S) and creep rate (m-value) of the asphalt samples, before and after the RTFOT aging, at −24 °C.

Figure 4 and Figure 5 represent the BBR experimental results for the asphalt samples stored at −18 °C and −24 °C, respectively. The addition of TPU appeared to reduce the creep stiffness (S) and improve the creep rate (m), indicating that the inner stress of the asphalt was decreased in the same temperature and loading condition and could also be lost, timely, through the deformation [22]. The improvement in these parameters indicated that the modified-asphalt performed better at a low-temperature and was not prone to cracking. However, when the asphalt samples aged, a series of volatilization, oxidation, polymerization, and even changes in the internal structure of the asphalt would have occurred and the asphalt would have become harder. The low-temperature flexibility was decreased, thus the creep stiffness was increased and creep rate was reduced. Consequently, the comprehensive analysis of S and the m-value, before and after the RTFOT aging, indicated that when the TPU content was 3%, the modified-asphalt had better short-term aging resistance than the other samples.

### 3.3. Thermal Properties

**Figure 6:**
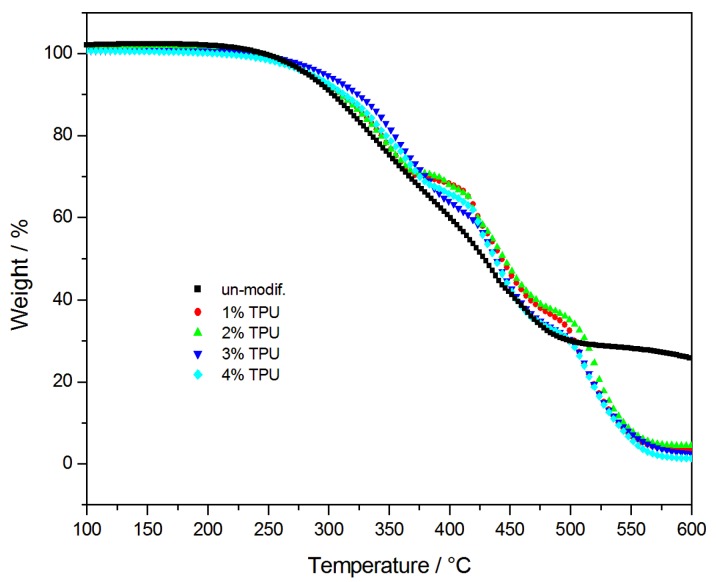
TG curves of base asphalt and modified-asphalts.

**Figure 7:**
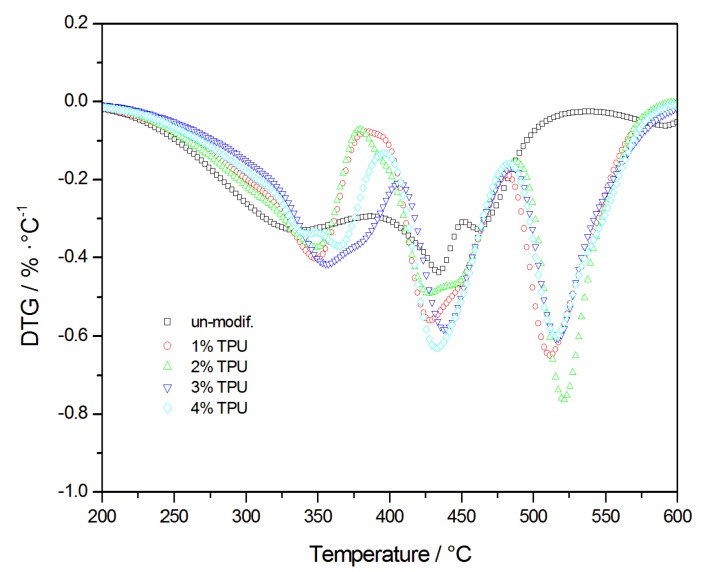
DTG curves of base asphalt and modified-asphalts.

The Thermal gravity (TG) and differential scanning calorimetry (DSC) curves of the asphalt combustion process are shown in Figure 6 and Figure 7. It is well-known that the components of the asphalt vary in chemical composition and molecular weight [1,23]. This leads to varying physical and chemical properties, in the asphalt, so that the temperature for mass loss of each component is different, consequently, there are several stages in asphalt degradation [24]. Every degradation stage of the asphalt combustion is listed in Table 1, based on Figure 7. The first degradation stage could mainly be attributed to the volatilization of the light-MW components of asphalt, such as the saturated and aromatic hydrocarbons. At this stage, the TPU in the modified-asphalt also began to decompose and absorbed heat, which delayed the time of the asphalt decomposition. With the increasing TPU content, the maximum degradation temperature of the modified-asphalt degradation increased and the thermal stability of the asphalt was improved. The most complex and intense chemical reaction occurred in the second-stage, where the asphalt mass loss was mainly due to the decomposition of resins. The thermal oxidative degradation of resins produced low molecular hydrocarbons that subsequently burned off. Dehydrogenation and polymerization occurred in the residual substance and increased the quantity of the carbonized products, while decreasing the stable free-radical content. The third stage was the degradation and the high-temperature charring of the asphaltene. In base asphalt, the release of volatiles and the combustion of fixed-carbon, rearranged the asphalt molecular structure and formed a dense layer of carbon. As a result, the oxygen and heat could not penetrate the unburned asphalt, which prevented the release of flammable volatiles that remained as a 25–26% of the residue. However, in the TPU-modified asphalt, the decomposition of the TPU caused the asphaltene to regain free radicals and the reactions continued. With an increasing temperature, the hydrogen and methyl of the asphaltene were continually removed and ultimately produced a stable char structure, the residual amount was 1–4%, and the mass-loss stopped. In addition, the derivative thermogravimetric (DTG) analysis curves of TPU-modified asphalt were flatter than the base asphalt’s curves, which suggested that the combustion activity of the TPU-modified asphalt had increased and its flame resistance had decreased.

### 3.4. Storage Stability

The segregation experiment results of the asphalt samples are shown in Table 2. In contrast to the conventional polymer-modified asphalt, the coalescence of modifying agents, during a high-temperature storage could not occur in the TPU-modified asphalt, which minimized their migration and the subsequent segregation. This was mainly due to the reaction between the –NCO groups in the TPU and the active hydrogen atoms (mainly –OH) in the asphaltene micelles [25], as shown below:

R^1^–NCO + R^2^–OH→R^1^–NHCOO–R^2^

Combined with the analysis of FTIR, in Section 3.6, the absorption peak at 2275 cm^−1^ was a characteristic peak of NCO. It was concluded that urethane bonds had formed between the polymer and the asphalt component (mainly asphaltenes and resins). Therefore, asphalt-rich phases had interacted together and formed a crosslinking. The modified-asphalt was uniform in its composition and highly dispersed, and a phase separation was avoided, during the high-temperature storage [26].

### 3.5. Morphology

**Figure 8:**
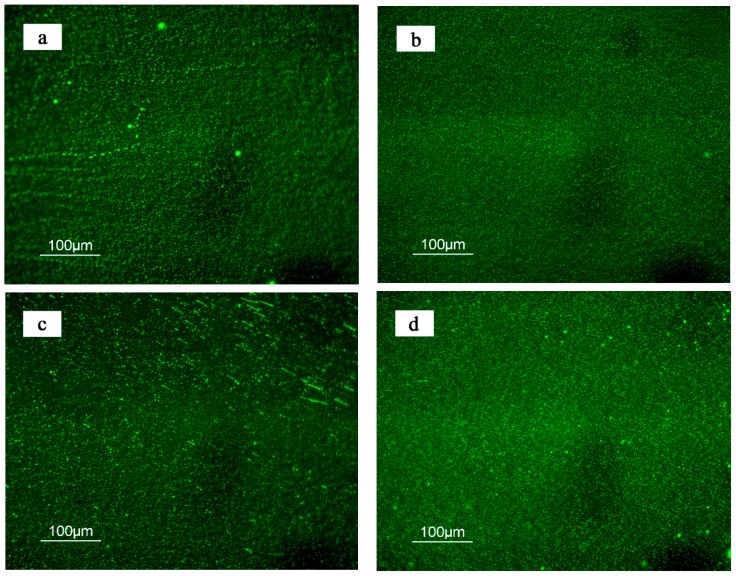
Morphology variation of asphalt samples, before and after aging (**a**: 2% TPU-modified asphalt; **b**: 2% TPU-modified asphalt, after the RTFOT aging; **c**: 4% TPU-modified asphalt; **d**: 4% TPU-modified asphalt, after the RTFOT aging.).

The morphology and dispersion of the TPU polymer in asphalt, before and after the short-term aging, is shown in Figure 8. In Figure 8a, the polymer appears as particles and is dispersed in a continuous phase in the asphalt. After the RTFOT aging, the quantity of the polymer particles had increased, their size had been reduced, and there was a lesser distinction between the polymer and the asphalt phases, as shown in Figure 8b. In Figure 8c, it appeared that the polymer content had increased, as had the polymer particles. The morphology of the 4% TPU-modified asphalt, after aging, is shown in Figure 8d, and it was similar to that of the 2% TPU-modified asphalt. As described above, the TPU could react with some of the asphalt components, making it easier for the TPU to achieve a more uniform dispersion in the asphalt system, than the polyethylene (PE), the SBS, and other non-reactive polymer-modifying agents [27,28]. In addition, the polymer chains are prone to fracture and decomposition in the aging process, thus, the compatibility of the polymer and the asphalt would be further improved and finally a more uniform modified asphalt system would be formed.

### 3.6. FTIR Analysis

**Figure 9:**
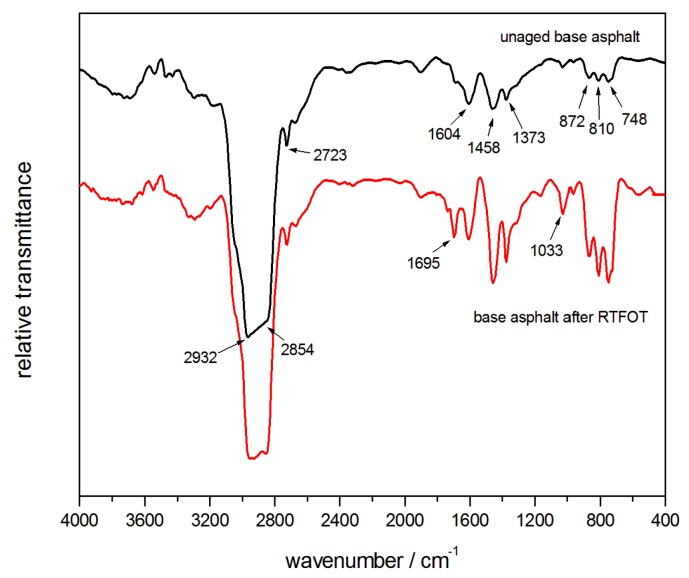
Infrared spectra of the base asphalt, before and after the RTFOT aging.

**Figure 10:**
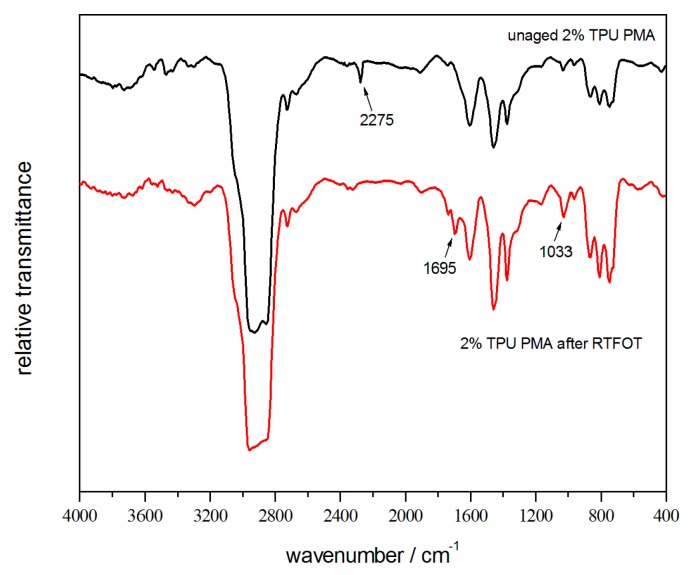
Infrared spectra of the modified-asphalt, before and after the RTFOT aging.

Figure 9 shows the infrared spectra of the base asphalt, before and after the short-term aging. The peaks at 2932 cm^−1^ and 2854 cm^−1^ were the typical stretching-vibrations of the aliphatic CH_2_. Aldehyde CH stretching-vibration was observed at 2723 cm^−1^ and the benzene ring vibration, C=C, was seen at 1604 cm^−1^. The peaks at 1458 cm^−1^ and 1373 cm^−1^ were assigned to the antisymmetric and symmetric deforming-vibrations of the CH_3_, respectively. Sulfoxide, S=O, stretching-vibration was observed at 1033 cm^−1^. The peaks at 872 cm^−1^, 810 cm^−1^, and 748 cm^−1^ were the stretching-vibrations (out-of-plane) of the C–H in phenyl [29,30]. After the RTFOT aging, a new peak at 1695 cm^−1^ appeared and the intensity of peak at 1033 cm^−1^ was enhanced. We attributed the presence of the carbonyl C=O to the thermal oxidation of the asphalt and the sulfoxides S=O, which resulted from the oxidation of sulfides in the asphalt. These were the characteristic peaks of asphalt-aging. In addition, the enhanced intensity of the peaks at 872 cm^−1^, 810 cm^−1^, and 748 cm^−1^ reflected the volatilization of the light alkane components and the relative increment of the fused-ring structured asphaltene, in the asphalt thermo-oxidative aging process. The infrared spectra of the modified-asphalt, before and after the RTFOT aging, could be summarized by the 2% TPU-modified asphalt shown in Figure 10. As described in Section 3.4, the characteristic peak of the NCO, at 2275 cm^−1^, had appeared. The remaining peaks, after the aging, were similar to that of the base asphalt, except that the characteristic peak at 1695 cm^−1^, was weaker than that of the base asphalt, indicating the role that was played by the reactive polymer TPU in the aging-resistance of the asphalt.

## 4. Conclusions

Compared with the base asphalt, the penetration ratio and the softening point increment of the TPU-modified asphalt was increased and decreased respectively, and continued to increase and decrease with the raised-TPU content, which confirmed that the aging resistance of the TPU-modified asphalt was enhanced. With respect to the combustion of the modified-asphalt, the degradation of the TPU absorbed the heat and delayed the decomposition of asphalt, thus, improving the asphalt thermal stability. TPU increased the thermal activity of the modified-asphalt and reduced its flame resistance. The weakened characteristic peak of the asphalt aging in the infrared spectrum also reflected the improvement in the aging-resistance of the TPU-modified asphalt. In general, crosslinking was formed and the asphalt-rich phase and the polymer-rich phase had interacted together, due to the reaction of the NCO reactive group in the TPU and the active hydrogen in the asphalt. Thus, the modified asphalt system could be stably stored and used. The fracture and degradation of the TPU chains, after the aging, further improved the compatibility of the polymer-modifying agent and the asphalt. TPU’s excellent properties could be reflected in the improvement of the modified-asphalt’s properties. The asphalt modification not only included the physical process but also included the chemical process, and further increased the comprehensive properties of the material.

## Figures and Tables

**Table 1:** Degradation stages in the asphalt combustion process.

Asphalt Samples	Combustion Process		
	Stage 1 (*T*_peak_)/°C	Stage 2 (*T*_peak_)/°C	Stage 3 (*T*_peak_)/°C
un-modif.	282–387 (337)	387–452(434)	452–536 (461)
1% TPU	283–383 (349)	383–482 (428)	482–575 (512)
2% TPU	286–379 (349)	379–488 (428)	488–577 (521)
3% TPU	297–407 (356)	407–485 (439)	485–578 (517)
4% TPU	284–396 (364)	396–483 (433)	483–580 (516)

**Table 2:** Softening point in the top and bottom segments of the asphalt samples.

Asphalt Samples	1% TPU	2% TPU	3% TPU	4% TPU
Softening point in top/°C	55.0	55.5	56.6	57.3
Softening point in bottom/°C	55.6	56.3	56.1	56.7
ΔSP ^a^	−0.6	−0.8	0.5	0.6

^a^ ΔSP means the difference in the softening point, in the top and the bottom segments, of the asphalt samples.
